# Efficacy of a plant-produced infectious bronchitis virus-like particle vaccine in specific pathogen-free chickens

**DOI:** 10.1016/j.psj.2023.102953

**Published:** 2023-07-22

**Authors:** Kamogelo M. Sepotokele, Martha M. O'Kennedy, Michaela C. Hayes, Daniel B.R. Wandrag, Peter Smith, Celia Abolnik

**Affiliations:** ⁎ Department of Production Animal Studies, Faculty of Veterinary Science, University of Pretoria, Pretoria, South Africa; † Next Generation Health Cluster, Council for Scientific and Industrial Research, Pretoria, South Africa

**Keywords:** *N. benthamiana*, infectious bronchitis, vaccine, virus-like particle

## Abstract

Infectious bronchitis (**IB**) Gammacoronavirus causes a highly contagious respiratory disease in chickens that is listed by the World Organisation for Animal Health (**WOAH**). Its high mutation ability has resulted in numerous variants against which the commercially available live or recombinant vaccines singly offer limited protection. *Agrobacterium*-mediated transient expression in *Nicotiana benthamiana* (tobacco) plants was used here to produce a virus-like particle (**VLP**) vaccine expressing a modified full-length IBV spike (**S**) protein of a QX-like IB variant. In a challenge study with the homologous live IB QX-like virus, VLP-vaccinated birds produced S protein-specific antibodies comparable to those produced by live-vaccinated birds seroconverting with mean geometric titers of 6.8 and 7.2 log_2_, respectively. The VLP-vaccinated birds had reduced oropharyngeal and cloacal viral shedding compared to an unvaccinated challenged control and were more protected against tracheal ciliostasis than the live-vaccinated birds. While the results appeared similar, plant-produced IB VLPs are safer, more affordable, easier to produce and update to antigenically match any emerging IB variant, making them a more suitable alternative to IBV control than live-attenuated vaccines.

## INTRODUCTION

Infectious bronchitis virus (**IBV**), listed by the World Organisation for Animal Health (**WOAH**) as a notifiable disease, causes extremely transmissible respiratory disease in chickens (*Gallus gallus*). IBV has an extensive economic impact on the global poultry industry, second only to that of avian influenza (World Bank & TAFS-Forum, 2011).

IBV is classified as a *Gammacoronavirus* of the *Coronaviridae* family, a single-stranded, enveloped RNA virus with a pleomorphic or sphere-shaped form, surrounded by club-shaped spikes (Jackwood and de Wit, 2013). The IBV spike (**S**) protein induces neutralizing antibodies (Kant et al., 1992; Jackwood and de Wit, 2013), but mutations occurring in the S protein S1 subunit result in the emergence of novel IBV serotypes that can evade protection offered by conventional serotype-specific vaccine types, such as live-attenuated or inactivated vaccines (Shirvani et al., 2018; Gallardo, 2021). At least 7 IBV genotypes exist, with over 35 different serotypes offering little or no cross-protection against antigenically distinct variants (Valastro et al., 2016), making IBV control exceedingly challenging with commercial serotype-specific vaccines (de Wit et al., 2011). However, in some cases the so-called “protectotype” phenomenon is useful, whereby combining 2 antigenically distinct IB vaccine serotypes confers broad serologic cross-protection against a third, unrelated serotype (de Wit et al., 2011; Butcher et al., 2022). For example, a combination of 4-91 and Mass-type variant vaccine strains is commonly used which has been shown to increase the level of protection against IBV in young chickens following challenge with different IBV variants (Terregino et al., 2008; Valastro et al., 2016). Elevated levels of protection can be achieved by priming with a live-attenuated vaccine prior to boosting with an inactivated vaccine or better yet, an inactivated autogenous variant vaccine (de Wit et al., 2011; Erfanmanesh et al., 2020). However, the production of autogenous variant vaccines normally entails the isolation and propagation of field virus in embryonated chicken eggs (Vaughn and Whitehead, 2009).

Virus-like particles (**VLP**) resemble native viruses, but lack the genetic material required to facilitate replication and cause infection (Ukarami et al., 2017). Several adaptations of VLPs against IBV have been developed in baculovirus expression vector systems, that could induce strong humoral and cellular immune responses in chickens, comparable to those elicited by inactivated IBV vaccines (Liu et al., 2013; Lv et al., 2014; Xu et al., 2016; Wu et al., 2019). Previously, a recombinant plant-produced IB VLP vaccine was successfully developed, with a modified full-length IBV S protein containing stabilizing proline mutations (Pallesen et al., 2017), and the following modifications: 1) the transmembrane domain (**TM**) and cytosolic tail (**CT**) of IBV substituted with that of a Newcastle disease virus (**NDV**) fusion protein (**F**), and 2) a murine signal peptide replacing the native signal peptide, coexpressed with the NDV matrix (**M**) protein (Sepotokele et al., 2023). A single immunization with the IB VLP vaccine was able to induce robust immune responses in specific pathogen-free (**SPF**) chickens after 2 wk (Sepotokele et al., 2023).

The present study further explored the protective efficacy of this IB VLP vaccine in SPF White Leghorn chickens, in a prime-boost strategy with live commercial vaccines that are known to confer protection against the QX-like variant used for challenge.

## MATERIALS AND METHODS

### Plant-Produced IB VLP Vaccine Production

The AGL-1 *Agrobacterium* stock containing the recombinant pEAQ-HT plasmid for the modified construct designated mIBV-S2P-NDV-F^TM/CT^ (Sepotokele et al., 2023) was propagated overnight at 28°C in Luria broth containing the selective antibiotics to an OD_600_ of ≤2. The recombinant plasmid for the NDV M protein, which had already been transformed into AGL-1, was propagated in the same way.

The overnight cultures were centrifuged at 10°C for 7 min at 7,000 × *g*, the pellet was resuspended in MES (2-(N-morpholino)ethanesulfonic acid) buffer, and diluted to an OD_600_ of between 1 and 1.5. The pEAQ-HT-mIBV-S2P-NDV-F^TM/CT^ construct was combined to a 4:1 ratio with pEAQ-HT-NDV-Matrix, and the mixture was left for 1 h at room temperature prior to infiltration. It was previously demonstrated that a ratio of 4:1 elevated the expression and assembly of IB VLPs (Sepotokele et al., 2023).

The leaves of 3- to 4-wk-old *Nicotiana benthamiana* ΔXT/FT plants (Strasser et al., 2008) were infiltrated with the prepared *Agrobacterium* culture combination using a needle-less syringe. Six days after agro-infiltration, the leaves were harvested, weighed and homogenized in a juicer in 2 volumes of either 1× Tris buffer (50 mM Tris base, 150 mM NaCl, pH 8.0), 1× Bicine buffer (20 mM NaCl, and 50 mM Bicine; pH 8.4) or 1× phosphate buffered saline (**PBS**) (140 mM NaCl, 1.5 mM KH_2_PO_4_, 10 mM Na_2_HPO_4_, 2.7 mM KCl, pH 7.4) containing protease inhibitor cocktail (Sigma-Aldrich, St. Louis, MO), and 0.04% sodium metabisulfite. The homogenized leaf extract was purified using sucrose density ultracentrifugation as previously described (Sepotokele et al., 2023).

Fractions 3 to 6 of the collected sucrose density gradient fractions were separated on a 12% sodium dodecyl sulfate-polyacrylamide gel electrophoresis (**SDS-PAGE**) gel and stained with Coomassie stain overnight. Western blots were performed with the same fractions using a 1:1,000 dilution of IBV antisera from commercial birds that had been vaccinated with live-attenuated Mass-type commercial vaccines. The secondary antibody used was a 1:2,000 dilution of Goat-α-Chicken IgY HRP (Abcam, Novex). Clarity Western ECL chemiluminescence substrate (BioRad, Hercules, CA) was used for protein detection on the ChemiDoc MP Imaging System (BioRad).

Carbon-coated holey copper grids of the plant-produced IB VLPs and SPF chicken egg allantoic fluid (**AF**) containing a live propagated IBV (strain ck/ZA/3665/11) were prepared as described previously (Sepotokele et al., 2023) and were imaged using a flash transmission electron microscope (**TEM**), JEOL JEM-1400, at the University of Pretoria.

The fractions containing the highest level of S protein expression as confirmed by SDS-PAGE and Western blot were pooled (i.e., those for mIBV-S2P-NDV-F^TM/CT^:Matrix processed in PBS buffer), dialyzed in 1× PBS using 3500 mW CO Slide-A-Lyzer Dialysis Cassettes (ThermoFisher Scientific, Waltham, MA), and stabilized with 15% (w/v) trehalose dehydrate (Sigma-Aldrich). The fractions containing the IB VLPs harvested in Tris and Bicine buffers were also dialyzed for comparison.

The partially purified VLPs were quantified using densitometric analysis. Bovine serum albumin (**BSA**) protein standards in known concentrations were electrophoretically separated alongside the dialyzed VLPs on a 12% SDS-PAGE gel. The overnight stained gel was analyzed using the ChemiDoc MP Imaging System Quantification Software (BioRad) with molecular weight markers and the protein standards serving as references.

### Vaccines and Challenge Virus Preparation

Live freeze-dried IB 4-91 and IB Ma5 vaccines (both sourced from Nobilis, MSD Animal Health, South Africa) were reconstituted separately in Diluvac (10 mL) (Nobilis, MSD Animal Health, South Africa) to the recommended single doses of ≥10^3.6^ and >10^3.5^ EID_50_, respectively; thereafter 1 mL of each was combined into 10 mL of Oculo Nasal Diluent (Nobilis, MSD Animal Health, Abnova, Kempton Park, South Africa).

The dialyzed VLPs were diluted to 20 µg (S protein content) per 225 µL with 1× PBS, and 10% (v/v) of EmulsigenP Adjuvant (MVP, Phibro Animal Health, IDEXX Laboratories Inc.: Omaha, NE) was added to a 250 µL volume.

Challenge virus strain ck/ZA/3665/11 (Bwala et al., 2018) was propagated in SPF chicken eggs and diluted in Diluent Oculo Nasal to an EID_50_ of 10^6^ per 60 µL, that is, 1 drop (30 µL) per eye of each bird. The vaccines and challenge virus were prepared on the day of use and kept at 4°C until administration.

### Animals

The vaccine efficacy challenge study was performed in isolators at the University of Pretoria's Biosafety Level 3 (**BSL3**). Three-week-old White Leghorn SPF chickens (*n* = 40; Avi-Farms (Pty.) Ltd., Pretoria, South Africa) were numbered individually with neck tags, and assigned randomly into 4 treatment groups, with 10 birds per isolator as follows: Group A: Live + VLP vaccinated; Group B: Live + Live vaccinated; Group C: Unvaccinated challenged control; Group D: Unvaccinated unchallenged control. Water and feed (Nova Feeds, Pretoria, South Africa) were provided ad libitum for the duration of the trial.

### Experimental Design

At d 0, 1 mL blood samples were taken from the wing veins of each bird prior to vaccination. Groups A and B were immunized with a single drop of the prepared live vaccine in each eye. Blood samples were collected again on d 21 from the birds in Groups A and B, before immunizing. Group B was again immunized via the intraocular route with another dose of mixed live vaccine, whereas the birds in Group A were immunized via inoculation in the breast with the 250 µL dose of the prepared VLP vaccine. On d 42, blood samples were collected from Groups A and B, prior to challenging. Groups A, B, and C were challenged via the intraocular route with the live QX-like virus. The birds were monitored for adverse vaccine effects daily throughout the trial, and for clinical signs postchallenge. Oropharyngeal and cloacal swabs were collected from all birds (Groups A, B, C, and D) on d 3, 5, and 7 post-challenge (**dpc**) using sterile rayon-tipped swabs with plastic applicators (Copan Diagnostics Inc., Murrieta, CA). The swabs were individually placed in 2 mL of viral transport media (0.1 mg/mL doxycycline, 1 mg/mL penicillin-streptomycin, brain-heart broth, 10% glycerol, 0.1 mg/mL enrofloxacin) and stored at 4°C prior to processing. Blood samples were taken again on d 49 (7 dpc) before euthanasia by cervical dislocation. The tracheas of each bird were removed directly after euthanasia, and placed into PBS for immediate ciliary motility scoring. The challenge model for strain ck/ZA/3665/11 whereby tracheas must be collected at d 7 pc for optimal vaccine efficacy evaluation was previously determined (Bwala et al., 2018).

### Serological Tests

Sera were harvested from clotted blood by low-speed centrifugation (3,000 × *g* at 22°C for 10 min) and tested using the commercial IDEXX IBV Antibody Kit (IDEXX Laboratories Inc.) following manufacturer's instructions. For the hemagglutination inhibition tests (**HI**), antigen was prepared by centrifuging AF from SPF eggs in which strain ck/ZA/3665/11 had been propagated to a titer of 10^6.5^ EID_50_/0.1 mL, for 15 min at 4°C. Five milliliters of the clarified AF was treated with 1 Unit of neuraminidase from *Clostridium perfringens* (P5290, Abnova, Taiwan) and incubated at 4°C overnight (Ruano et al., 2000). The HI tests were performed with 1% (v/v) chicken red blood cells (**CRBC**) as per the recommended method of the WOAH (WOAH, 2019), but at room temperature. The last well in which CRBC streaming was observed in a 2-fold titration of the test serum relative to the controls was recorded as the log_2_ HI titer.

### Quantification of QX-Like IBV

Total nucleic acid was extracted from 0.2 mL of the swab fluids using the IndiMag Pathogen Kits as recommended in the IndiMag 48 instrument (Indical BioSciences, Leipzig, Sachsen, Germany). The presence of QX-like IBV-specific RNA was detected using the Vetmax-Plus One Step RT-PCR kit (Applied Biosystems, Waltham, MA) in a StepOnePlus instrument (Applied Biosystems) as described in Bwala et al. (2018). This assay was designed and shown to specifically detect only the S-protein gene sequence of the QX-like variant, but not any other serotypes, including the 4-91 and Ma5 vaccine strains. The qRT-PCR reactions included 0.15 µL of the QX-like-specific IBV MGB-FAM-labeled probe (5 pmol/µL), 4 µL of extracted RNA, 6 µL of 2× RT-PCR buffer, 0.5 µL each of IBV reverse and forward primer (12.5 pmol/µL), 0.5 µL of 25× RT-PCR enzyme mix, and nuclease-free water to make the volume up to 13 µL. The RT-PCR cycling profile was as follows: 10 min at 48°C, 10 min at 95°C, and forty cycles of 15 s at 95°C plus 45 s at 60°C. Test samples were run alongside a standard curve prepared from the titrated ck/ZA/3665/11 stock and no-template negative controls. The limit of detection of the qRT-PCR assay was <15.93 EID_50_ equivalents/mL (results of this study).

### Ciliary Motility Scoring

Immediately after the birds were euthanized, their tracheas were removed and submerged in 10 mL of 1× PBS in 15 mL Universal tubes. The tracheas were cut into 10 ring sections, 3 from the upper, 4 from the mid-section and 3 from the lower section, floated in drops of 1× PBS in a demarcated Petri dish, and analyzed for ciliary motility across the circumference of the tracheal section under a light microscope at 10× magnification. Each of the 10 rings for each bird was given a percentage correlating to a score ranging from 0 to 4 as follows: 0: 75 to 100% motility (normal); 1: 51 to 75% motility; 2: 30 to 50% motility; 3: 3 to 30% motility; 4: 0 to 2% motility (little to no motility). The average protection score for each group was calculated using the following equation (Andrade et al., 1982):

$$\text{Average}\;\text{Protection}\;\text{Score}\;=\;100-\frac{\text{sum}\;\text{total}\;\text{of}\;\text{individual}\;\text{scores}\;\text{in}\;\text{group}}{\text{total}\;\text{number}\;\text{of}\;\text{individuals}\;\text{within}\;\text{group}\;\times \;20}\;\times \;100$$

### Statistical Analysis

GraphPad Prism v 9.4.1 software for Windows (La Jolla, CA) was used for statistical analysis. The results for the HI tests were compared statistically by means of the unpaired *T* test and a *P* value less than 0.05 was considered significant. The results of the qRT-PCR reactions were analyzed by means of 1-way analysis of variance (**ANOVA**) followed by the Tukey's multiple comparisons test. The tests were performed on the untransformed data, with the results presented as log_10_ values for ease of comparison.

## RESULTS

### Production of the VLP Vaccine in Plants

**Figure 1:**
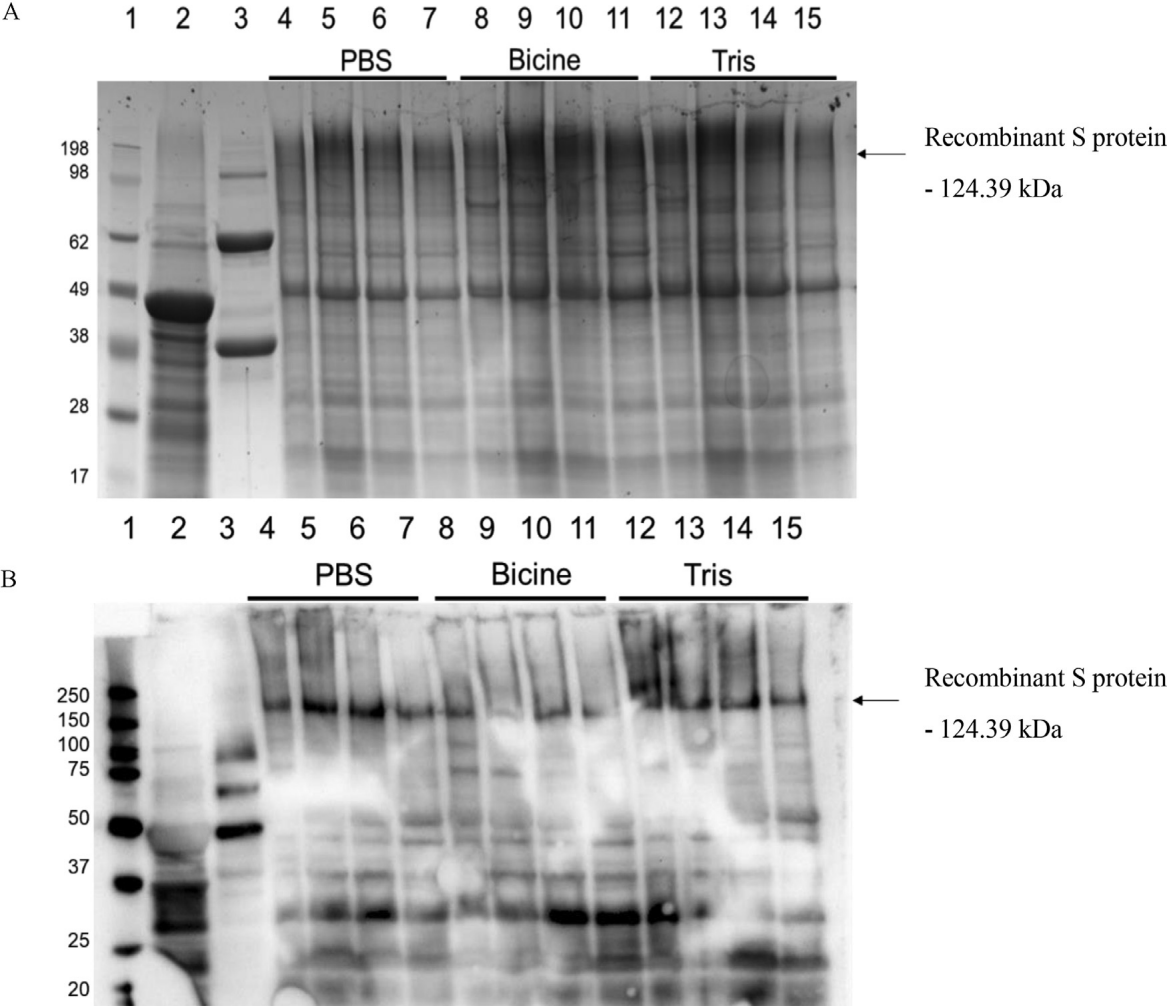
SDS-PAGE (A) and Western blot (B) of plant-produced spike protein (synthetic construct mIBV-S2P-NDV-F^TM/CT^) coinfiltrated at a 4:1 ratio with the NDV matrix protein. Lane 1: molecular weight marker; Lane 2: plant-expressed empty pEAQ-HT vector; Lane 3: purified live QX-like IBV strain ck/ZA/3665/11; Lanes 4 to 7: Fractions 3 to 6 extracted in PBS buffer; Lanes 8 to 11: Fractions 3 to 6 extracted in Bicine buffer; Lanes 12 to 15: Fractions 3 to 6 extracted in Tris buffer.

The leaves of agroinfiltrated tobacco plants were harvested after 6 d, and the IB VLP yields obtained through purification in PBS, Tris, or Bicine buffer were compared. Analysis by SDS-PAGE and immunoblot with IBV antisera confirmed S protein expression with a 124 kDa band correlating to the expected size (Figure 1). The immunoblot showed stronger S protein-specific detection with the antisera in the samples where PBS was used for extraction than in the samples where either Bicine or Tris was used (Figure 1B). The QX-like antisera did not detect a band correlating to the size of the S protein in the positive control (purified live virus), although the flock from which the antisera was collected had been immunized with IBV vaccines of the Mass serotype only, and the level of cross-protection seen between single serotypes is typically low (Cavanagh, 2003).

**Figure 2:**
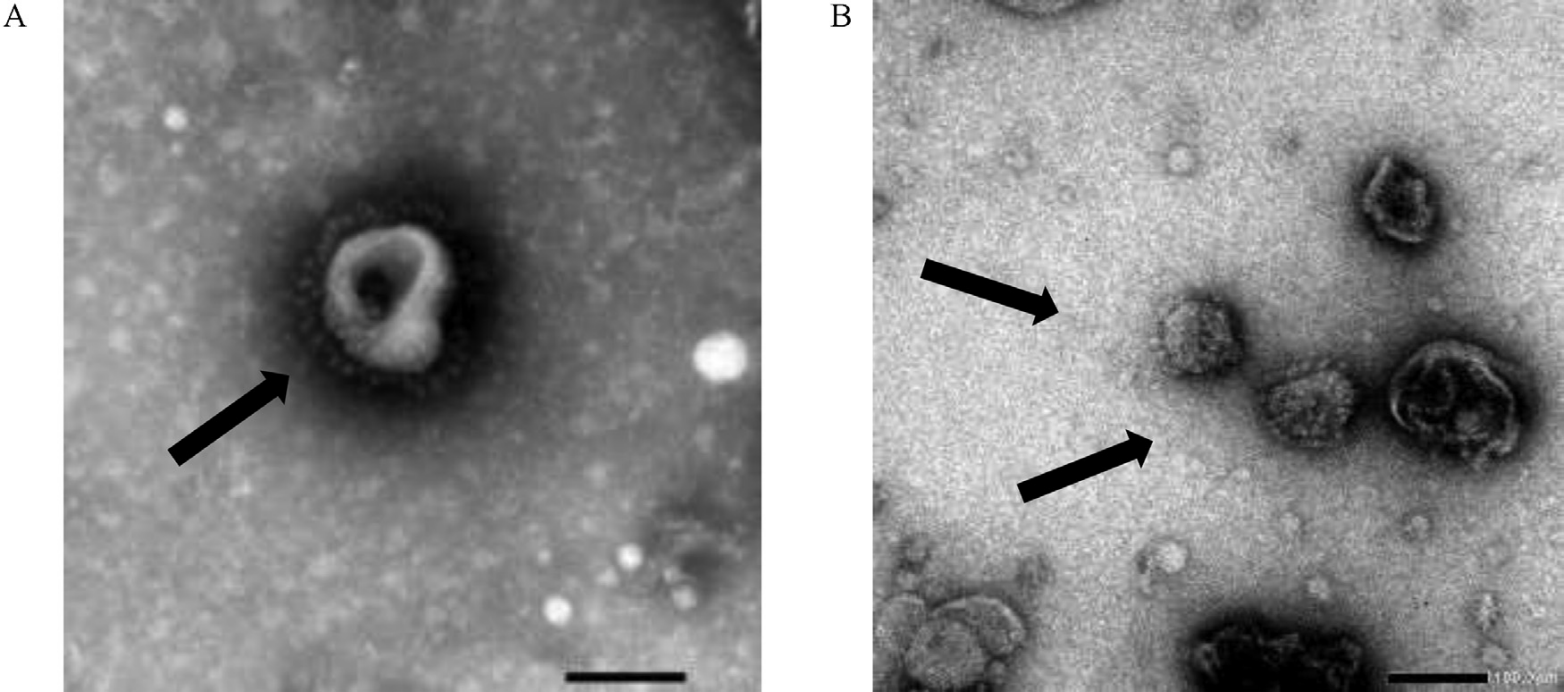
Negative-stained transmission electron microscopy images of (A) live QX-like IB virus strain ck/ZA/3665/11 and (B) plant-produced IBV virus-like particles displaying the spike protein expressed with the synthetic gene construct mIBV-S2P-NDV-F^TM/CT^ in *N. benthamiana* in different ratios with NDV matrix. Arrows indicate VLPs.

IB VLPs extracted in either Bicine or Tris buffer yielded no VLPs when analyzed by TEM (data not shown), while the sample harvested with PBS contained VLPs that resembled typical IBV particles in morphology and size, ranging from 67 to 135 nm in diameter (Figure 2). The dialyzed samples from all 3 extraction methods were analyzed using densitometry, but the S protein was undetectable in the samples extracted with either Bicine or Tris, while the sample extracted in PBS had an S protein concentration of approximately 67 ng/µL for the S protein band. The latter sample was therefore used to prepare the vaccine in the efficacy study. Unlike plant-produced avian influenza VLPs (Smith et al., 2020), hemagglutination (**HA**) could not be used to quantify the IB VLPs because they do not agglutinate chicken red blood cells (Sepotokele et al., 2023).

### Efficacy Study

#### Clinical Signs

There were no observable clinical signs in any of the birds, including the nonvaccinated challenged controls in group C, but the lack of clinical signs was consistent with previous results in SPF chickens infected with strain ck/ZA/3665/11 (Bwala et al., 2018). No adverse vaccine effects were reported for any of the birds following immunization throughout the trial.

#### Immunogenicity in Chickens of IB Vaccines Pre- and Postchallenge

As expected, no IB-specific antibodies were detected prior to vaccination (Table S1), and after 21 d, birds in Groups A and B immunized with a mix of the live 4-91/Mass type vaccines, tested positive for the presence of IBV antibodies with average antibody titers of 3347 and 2903, respectively. Twenty-one days later, after birds were boosted with the VLP vaccine (Group A) or with the live vaccine mix (Group B), only marginal increases in the IBV-specific antibodies were detected, with average antibody titers of 3354.49 and 2911, respectively.

**Figure 3:**
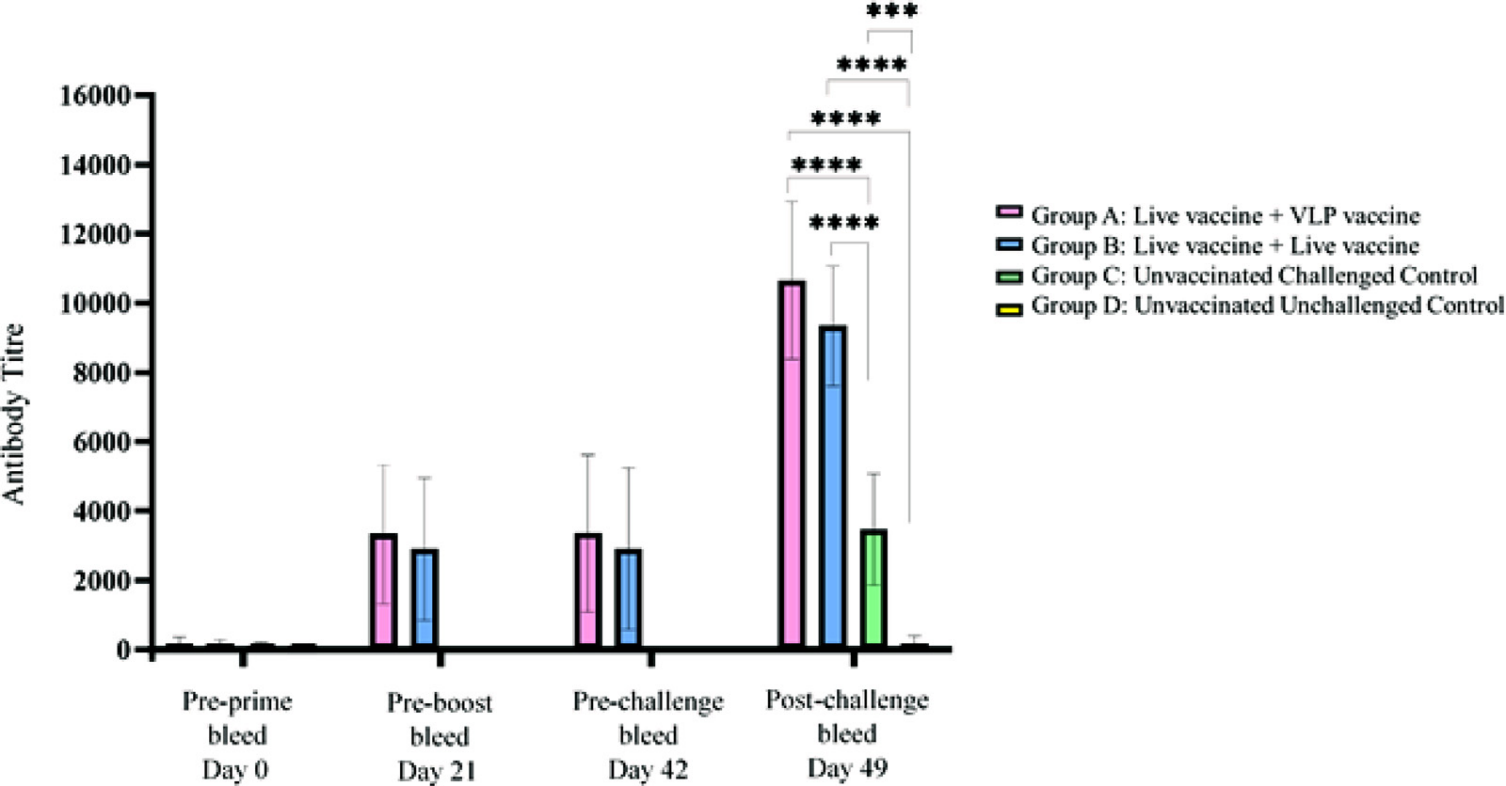
IDEXX IB ELISA antibody titers of SPF chickens vaccinated with combinations of mixed live and VLP vaccines, and challenged with live QX-like IB virus, and controls. The asterisks indicate the statistical significance *** (*P* ≤ 0.001); **** (*P* ≤ 0.0001). Only significant differences are indicated on the graph (*P* ≤ 0.05).

Seven days after challenge, seroconversion was evident, where the average antibody titers of Group A were descriptively but not significantly higher (10660.74) than Group B (9344), but both vaccinated groups had significantly higher IBV-specific antibodies than Group C, the unvaccinated challenged control (3471.02) (Figure 3).

**Figure 4:**
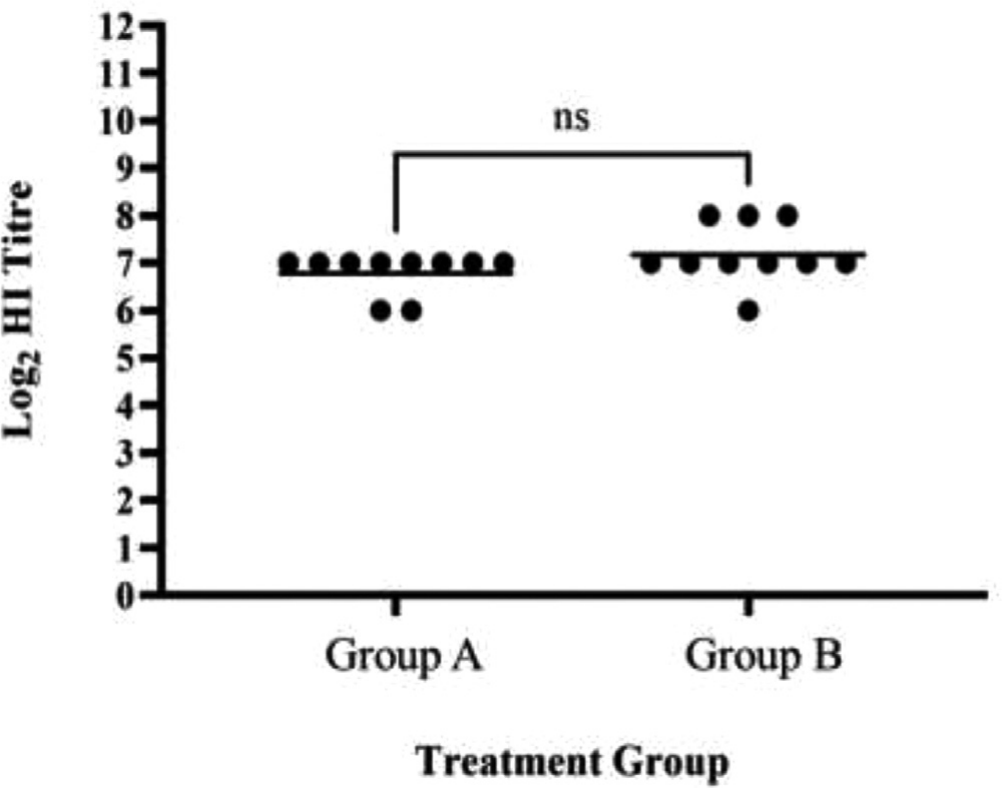
Hemagglutination inhibition (HI) log_2_ titers for the chicken sera in Groups A (Live + VLP vaccinated) and B (Twice Live vaccinated) taken on d 42 (prechallenge). The bar shows the geometric mean titer; ns, not significant (*P* ˃ 0.05).

The prechallenge antisera (d 42) were also tested by HI, and Group A exhibited HI titers that ranged from 6 to 7 log_2_ (geometric mean titer (**GMT**) of 6.8 log_2_), while Group B exhibited HI titers that ranged from 6 to 8 log_2_ (GMT of 7.2 log_2_) (Table S1), but there was no statistical difference between the GMTs (Figure 4).

#### Ability of Vaccines to Reduce IB Virus Shedding

**Figure 5:**
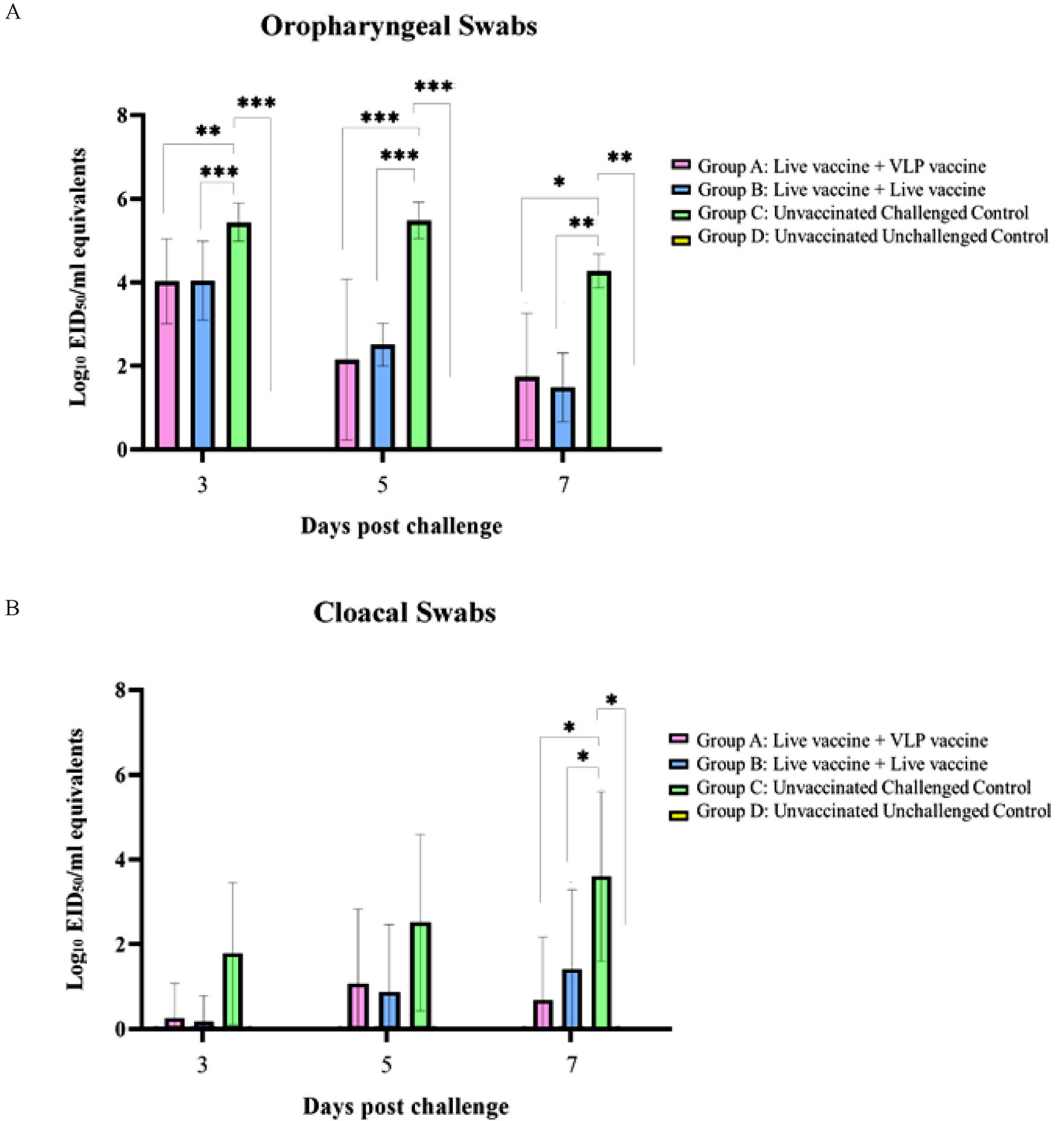
rRT-PCR detection of QX-like IB virus in the (A) oropharyngeal and (B) cloacal swabs taken at 3-, 5-, and 7-days postchallenge. The asterisks indicate the statistical significance * (*P* ≤ 0.05), ** (*P* ≤ 0.01), *** (*P* ≤ 0.001), **** (*P* ≤ 0.0001). Only significant differences are indicated on the graph (*P* ≤ 0.05).

The qRT-PCR assay was designed to detect QX-like IBV RNA of the challenge virus only, and not the presence of any residual live vaccines. Prime-boost vaccination with either the live mixed vaccine and VLP (Group A) or only live mixed vaccines (Group B) caused progressive and statistically significant reduction in virus shedding from the oropharynx of infected birds at d 3, 5, and 7 postchallenge in comparison to the nonvaccinated control birds (Group C) (Figure 5A; Table S2). The levels of virus shedding between vaccinated groups A and B were comparable with no statistical difference between the means. By d 7 postchallenge, the vaccines had reduced the mean virus shedding from the respiratory tract to below 1.8 log_10_ EID_50_/mL equivalents, whereas the nonvaccinated challenge control group C still shed high virus titers with a mean of 4.27 log_10_ EID_50_/mL equivalents and would therefore continue to shed virus from the respiratory tract for a longer period than the vaccinated chickens.

Similarly, both vaccination regimes in groups A and B reduced virus shedding from the cloaca in comparison to the nonvaccinated control group C after challenge, but the difference was only statistically significant at d 7 postchallenge (Figure 5B; Table S2). Challenge virus shedding from the cloaca continued to increase in group C up until d 7, where it reached a mean titer of 3.44 log_10_ EID_50_/mL equivalents. Interestingly, it appears that virus shedding from the cloaca in birds boosted with the VLP peaked at d 5 postchallenge at 0.99 log_10_ EID_50_/mL equivalents before dropping to 0.67 log_10_ EID_50_/mL equivalents at d 7 postchallenge, whereas the levels of cloacal shedding in birds boosted with the live vaccines (Group B) continued to increase up until d 7 postchallenge, reaching a mean titer of 1.36 log_10_ EID_50_/mL equivalents, therefore it is possible that the endpoint for virus shedding from the cloaca would be reached later for group B compared to group A. The likely reason for this is the antigenic match between the VLP and the challenge virus.

#### Effect of Vaccines on Ciliary Motility

The ciliary motility scoring indicated that both vaccination regimes (Groups A and B) provided 100% protection overall in comparison to 0% in the nonvaccinated challenged group (C) (Figure S3), but there were differences in the average protection scores for groups A and B of 79 and 67, respectively. This indicates that the VLP-boosted group A provided slightly better protection against the virus-induced depletion of the tracheal cilia than group B that only received the heterologous live vaccines.

## DISCUSSION

Live-attenuated vaccines elicit robust local immune responses which offer protection against respiratory tract infection that is maintained at high levels for prolonged time periods, such as throughout the laying period of hens (Jackwood and de Wit, 2013). Theoretically, complete protection against IBV infection is usually dependent on the administration of the homologous vaccine; the protection conferred by heterologous vaccines ranges from very poor to moderate protection, depending on the assigned criteria of protectotyping (Hassan et al., 2021). Protectotype vaccination is useful in some cases where 2 vaccine serotypes may be applied that offer heterologous cross-protection against a wider range of IBV strains (Butcher et al., 2022).

Inactivated autogenous or variant vaccines (such as virus-like particles), however, have been developed from new variant strains of IBV, which are used as an alternative to live vaccines for controlling the spread of the virus in laying birds (Jackwood and de Wit, 2013). Variant vaccines have the advantage of being highly antigenically matched to variant IBV strains that may be circulating in a particular region. They are able to provide superior protection against virulent variant IBV strains than vaccines traditionally produced using the more commonly used Massachusetts or Connecticut serotypes (Ladman et al., 2002).

Previously, we successfully produced VLPs displaying the IBV S protein in tobacco plants for the first time, where the S protein, modified with 2 stabilizing proline residues, expressed optimally in VLPs with the substituted TM and CT of NDV, coexpressed with NDV M protein (Sepotokele et al., 2023). In this study, we produced a VLP batch and evaluated the efficacy of this plant-produced vaccine against homologous challenge with a QX-like virus. The use of a live-attenuated virus for the prime dose during vaccination improves the level of protection against IBV, while using inactivated vaccines offered broad-spectrum protection against multiple strains of IBV (de Wit et al., 2022). Therefore, the plant-produced VLP was applied as a booster 3 wk after vaccination with a mix of live commercial 4-91 and Ma5 IB vaccines, as simultaneous or alternate use of Ma5 and 4/91, as commonly employed in many countries, including South Africa, induces high levels of protection against heterologous IBV types such as QX (Cook et al., 1999; Terregino et al., 2008). For comparison, chickens primed and boosted with the Ma5 and 4/91 mix were included as a control group.

Prior to challenge, both vaccination regimes appeared to provide similar humoral protection as measured by enzyme-linked immunosorbent assay (**ELISA**) and HI, but like all commercially available IB ELISAs, the IDEXX IB Ab ELISA is not a serotype-specific assay, and is validated to detect antibodies directed against more conserved structural IBV nucleocapsid (**N**) and membrane (**M**) proteins. The plant-produced VLP does not contain any IB proteins apart from S, therefore the commercial ELISAs probably underestimated the S-specific antibodies induced by the VLPs. The HI results, which specifically measure S-protein antibodies, were also comparable between the VLP and live vaccines, but once there is multiple exposure to IBV, as with prime-boost vaccination, the HI test displays a variety of cross-reactions with other IBV serotypes that are not homologous to the HI test antigen, creating the challenge of being unable to differentiate between varying serotypes (Cook et al., 1987; Gelb and Jackwood, 2008).

Since the QX strain 3665/11 does not induce clinical signs (e.g., respiratory signs or kidney pathology) under experimental conditions in SPF chickens (Bwala et al., 2018), we were limited in assessing the vaccine efficacy on the reduction in viral shedding and ciliary motility.

Overall, the protection against challenge was similar, with no statistically significant difference in the ability of the vaccines to reduce the magnitude of virus shedding from the oropharynx or cloaca, but boosting with the VLP vaccine did seem to reduce the duration of cloacal shedding more effectively, which in turn would aid in reducing fomite spread of IBV between flocks. The VLP vaccine booster also had an improved average protection score on the tracheal ciliary motility test, suggesting that boosting with the VLP vaccine offered better respiratory tract protection than vaccinating with the heterologous 4/91 and Ma5 vaccine mix alone. Respiratory tract protection is vital in field conditions where flocks are likely to be exposed to several pathogens. It is a limitation of this study that we used a vaccine combination for which a protectotype is available.

Ultimately, the benefit of plant-produced homologous IB VLPs over traditional whole inactivated virus vaccines is the safety, speed and scalability with which they can be produced. Bulk VLP doses can be produced in as little as 2 wk after obtaining the S gene sequence (Lai and Chen, 2012) and although there is still scope to improve the yields, in this case 1 kg of plant leaf material was sufficient to produce at least 3,352 individual 5 µg VLP vaccine doses. Like inactivated vaccines, VLP vaccines require intramuscular administration by injection, but there is the option of exploring other delivery methods such as formulated inhalable powders for mucosal immunization for the mass vaccination of flocks (Tomar et al., 2018). In conclusion, plant-produced IB VLPs have incredible potential to improve poultry health providing the ability to develop safe homologous IBV vaccines quickly and affordably, scaling them up to meet agricultural requirements of poultry industries even in lower income parts of the world, making it possible to effectively deal with the persistent global issue of emerging IBV variants.

## Appendix Supplementary materials

Image, application 1

## Footnotes

Supplementary material associated with this article can be found in the online version at doi:10.1016/j.psj.2023.102953.
