# Supplementary material for: Efficacy of a plant-produced infectious bronchitis virus-like particle vaccine in specific pathogen-free chickens
Source: Poult Sci. 2023 Jul 22;102(10):102953. doi: 10.1016/j.psj.2023.102953 (PMC10407904; doi:10.1016/j.psj.2023.102953)
Supplement: Supplementary file 1 [file mmc1.docx]

**Table S1. Serology test results**

|  |  | **Day 0** | | **Day 21** | | **Day 42** | | | **Day 49** | |
| --- | --- | --- | --- | --- | --- | --- | --- | --- | --- | --- |
|  |  | **Pre-prime** | | **Pre-boost** | | **Pre-challenge** | | | **Post challenge** | |
|  |  | **ELISA** | | **ELISA** | | **ELISA** | | **HI Log_2_ titre** | **ELISA** | |
| **Treatment Group** | **Bird no.** | **S/P** | **Titre** | **S/P** | **Titre** | **S/P** | **Titre** |  | **S/P** | **Titre** |
| **Group A:**  **Live vaccine + VLP vaccine** | 10241 | 0.02 | 61.51 | 0.86 | 3906.55 | 1.39 | 6592.89 | 6 | 2.67 | 13421.64 |
|  | 10244 | 0.13 | 514.77 | 1.09 | 5024.36 | 1.43 | 6765.41 | 6 | 2.51 | 12507.8 |
|  | 10245 | 0.02 | 61.51 | 0.31 | 1269.57 | 0.11 | 401.55 | 7 | 2.07 | 10148.45 |
|  | 10246 | 0.02 | 72.76 | 0.61 | 2686.58 | 0.72 | 3202.47 | 7 | 2.73 | 13744.42 |
|  | 10247 | 0.03 | 84.16 | 1.67 | 8035.35 | 1.17 | 5464.02 | 7 | 1.93 | 9387.99 |
|  | 10248 | 0.02 | 72.76 | 0.85 | 3857.06 | 0.61 | 2670.58 | 7 | 2.14 | 10513.84 |
|  | 10249 | 0.01 | 18.57 | 0.62 | 2750.65 | 0.61 | 2670.58 | 7 | 1.73 | 8354.76 |
|  | 10250 | 0.02 | 61.51 | 0.55 | 2384.01 | 0.75 | 3365.17 | 7 | 2.32 | 11498.87 |
|  | 10252 | 0.08 | 291.41 | 0.51 | 2210.25 | 0.42 | 1773.21 | 7 | 2.15 | 10597.04 |
|  | 10254 | 0.12 | 459.95 | 0.32 | 1344.96 | 0.16 | 638.91 | 7 | 1.36 | 6432.64 |
|  | **GMT:** | **0.05** | **169.89** | **0.74** | **3346.93** | **0.74** | **3354.48** | **6.8** | **2.16** | **10660.74** |
| **Group B:**  **Live vaccine + Live vaccine** | 10264 | 0.04 | 130.93 | 0.46 | 1990.74 | 0.73 | 3251.21 | 8 | 1.83 | 8861.93 |
|  | 10265 | 0.01 | 18.57 | 0.57 | 2479.23 | 0.53 | 2289.09 | 7 | 1.83 | 8878.33 |
|  | 10267 | 0.01 | 39.54 | 0.2 | 811.22 | 0.29 | 1194.55 | 8 | 1.66 | 7963.84 |
|  | 10270 | 0.03 | 107.34 | 1.13 | 5243.81 | 0.46 | 1990.74 | 7 | 2.3 | 11415.12 |
|  | 10272 | 0.05 | 166.99 | 0.93 | 4254.38 | 1.9 | 9251.24 | 8 | 2.41 | 11968.84 |
|  | 10274 | 0.11 | 420.51 | 0.33 | 1375.21 | 0.63 | 2766.68 | 7 | 2.2 | 10880.29 |
|  | 10276 | 0.08 | 304.14 | 0.38 | 1588.48 | 0.43 | 1835.14 | 7 | 1.31 | 6177.51 |
|  | 10277 | 0.03 | 84.16 | 0.74 | 3300.01 | 0.62 | 2718.6 | 7 | 1.98 | 9651.94 |
|  | 10279 | 0.05 | 179.16 | 0.23 | 913.19 | 0.26 | 1045.69 | 7 | 1.72 | 8289.5 |
|  | 10280 | 0.05 | 191.4 | 1.49 | 7076.85 | 0.63 | 2766.68 | 6 | 1.92 | 9355.03 |
|  | **GMT:** | **0.05** | **164.27** | **0.65** | **2903.31** | **0.65** | **2910.96** | **7.2** | **1.92** | **9344.23** |
| **Group C:**  **Unvaccinated challenged control** | 10281 | 0.01 | 39.54 | NT | NT | NT | NT | NT | 0.37 | 1536.22 |
|  | 10282 | 0.02 | 72.76 | NT | NT | NT | NT | NT | 0.94 | 4309.58 |
|  | 10283 | 0.02 | 72.76 | NT | NT | NT | NT | NT | 1.14 | 5291.62 |
|  | 10284 | 0.02 | 61.51 | NT | NT | NT | NT | NT | 1.16 | 5417.47 |
|  | 10285 | 0.11 | 433.63 | NT | NT | NT | NT | NT | 1.02 | 4681.72 |
|  | 10286 | 0.02 | 61.51 | NT | NT | NT | NT | NT | 0.79 | 3543 |
|  | 10287 | 0.01 | 28.89 | NT | NT | NT | NT | NT | 0.6 | 2626.4 |
|  | 10288 | 0.01 | 28.89 | NT | NT | NT | NT | NT | 0.13 | 498.25 |
|  | 10289 | 0.02 | 50.42 | NT | NT | NT | NT | NT | 0.88 | 3986.11 |
|  | 10290 | 0.01 | 39.54 | NT | NT | NT | NT | NT | 0.64 | 2819.8 |
|  | **GMT:** | **0.03** | **88.94** |  |  |  |  |  | **0.77** | **3471.02** |
| **Group D:**  **Unvaccinated unchallenged control** | 10291 | 0.04 | 119.09 | NT | NT | NT | NT | NT | -0.01 | 0 |
|  | 10292 | 0.02 | 50.42 | NT | NT | NT | NT | NT | 0.02 | 81.75 |
|  | 10293 | 0.03 | 95.69 | NT | NT | NT | NT | NT | 0.17 | 655.26 |
|  | 10294 | 0.03 | 107.34 | NT | NT | NT | NT | NT | 0.11 | 433.95 |
|  | 10295 | 0.02 | 72.76 | NT | NT | NT | NT | NT | 0.02 | 48.98 |
|  | 10296 | 0.03 | 107.34 | NT | NT | NT | NT | NT | 0.03 | 104.26 |
|  | 10297 | 0.03 | 95.69 | NT | NT | NT | NT | NT | 0.02 | 70.67 |
|  | 10298 | 0.02 | 61.51 | NT | NT | NT | NT | NT | 0.03 | 92.95 |
|  | 10299 | 0.02 | 50.42 | NT | NT | NT | NT | NT | 0.08 | 283.05 |
|  | 10300 | 0.01 | 39.54 | NT | NT | NT | NT | NT | -0.01 | 0 |
|  | **GMT:** | **0.02** | **79.98** |  |  |  |  |  | **0.05** | **177.09** |

S/P- sample: positive; HI- hemagglutinin inhibition; NT- not tested; GMT- geometric mean titre

**Table S2. Detection of QX-like IBV using quantitative real-time reverse transcription PCR**

|  |  | **Oropharyngeal swabs**  **Log_10_ EID_50_/ml** | | | **Cloacal swabs**  **Log_10_ EID_50_/ml** | | |
| --- | --- | --- | --- | --- | --- | --- | --- |
| **Treatment Group** | **Bird no.** | **3 dpc** | **5 dpc** | **7 dpc** | **3 dpc** | **5 dpc** | **7 dpc** |
| **Group A: Live vaccine + VLP vaccine** | 10241 | 2.25 | 0 | 3.06 | 0 | 0 | 0 |
|  | 10244 | 3.72 | 0 | 2.90 | 0 | 0 | 0 |
|  | 10245 | 5.01 | 3.96 | 3.37 | 0 | 2.95 | 0 |
|  | 10246 | 2.58 | 0 | 0 | 0 | 3.26 | 0 |
|  | 10247 | 4.88 | 4.40 | 0 | 0 | 0 | 0 |
|  | 10248 | 3.86 | 3.13 | 0 | 0 | 3.72 | 3.24 |
|  | 10249 | 4.20 | 2.67 | 0 | 2.46 | 0 | 3.47 |
|  | 10250 | 5.23 | 4.19 | 2.93 | 0 | 0 | 0 |
|  | 10252 | 4.84 | 3.17 | 2.76 | 0 | 0 | 0 |
|  | 10254 | 3.70 | 0 | 2.40 | 0 | 0 | 0 |
|  | **Ave:** | **4.03** | **2.15** | **1.74** | **0.25** | **0.99** | **0.67** |
| **Group B: Live vaccine + Live vaccine** | 10264 | 4.96 | 2.44 | 1.74 | 0 | 0 | 0 |
|  | 10265 | 3.94 | 3.05 | 2.06 | 0 | 0 | 3.61 |
|  | 10267 | 4.23 | 2.69 | 0 | 0 | 0 | 0 |
|  | 10270 | 4.72 | 2.36 | 1.63 | 0 | 4.20 | 3.85 |
|  | 10272 | 4.06 | 2.48 | 0 | 1.80 | 1.66 | 0 |
|  | 10274 | 4.50 | 2.02 | 1.50 | 0 | 0 | 0 |
|  | 10276 | 1.87 | 2.17 | 2.29 | 0 | 0 | 0 |
|  | 10277 | 3.36 | 2.04 | 1.79 | 0 | 0 | 2.91 |
|  | 10279 | 5.11 | 3.66 | 2.13 | 0 | 0 | 0 |
|  | 10280 | 3.68 | 2.18 | 1.74 | 0 | 2.28 | 3.26 |
|  | **Ave:** | **4.04** | **2.51** | **1.49** | **0.18** | **0.81** | **1.36** |
| **Group C: Unvaccinated Challenged Control** | 10281 | 5.73 | 5.48 | 4.31 | 1.20 | 1.64 | 3.27 |
|  | 10282 | 5.39 | 5.47 | 4.53 | 0 | 3.80 | 4.62 |
|  | 10283 | 5.46 | 4.70 | 4.39 | 5.05 | 1.50 | 3.66 |
|  | 10284 | 4.56 | 6.03 | 3.90 | 2.73 | 0 | 0 |
|  | 10285 | 6.16 | 6.13 | 5.14 | 2.34 | 1.65 | 4.75 |
|  | 10286 | 5.44 | 5.18 | 4.41 | 2.60 | 1.63 | 0 |
|  | 10287 | 4.82 | 5.18 | 4.22 | 1.54 | 0 | 5.22 |
|  | 10288 | 5.62 | 5.22 | 4.26 | 0 | 2.31 | 4.29 |
|  | 10289 | 5.70 | 5.70 | 3.85 | 1.45 | 5.41 | 4.09 |
|  | 10290 | 5.50 | 5.76 | 3.75 | 0 | 5.15 | 4.50 |
|  | **Ave:** | **5.44** | **5.48** | **4.27** | **1.69** | **2.31** | **3.44** |
| **Group D: Unvaccinated Unchallenged Control** | 10291 | 0 | 0 | 0 | 0 | 0 | 0 |
|  | 10292 | 0 | 0 | 0 | 0 | 0 | 0 |
|  | 10293 | 0 | 0 | 0 | 0 | 0 | 0 |
|  | 10294 | 0 | 0 | 0 | 0 | 0 | 0 |
|  | 10295 | 0 | 0 | 0 | 0 | 0 | 0 |
|  | 10296 | 0 | 0 | 0 | 0 | 0 | 0 |
|  | 10297 | 0 | 0 | 0 | 0 | 0 | 0 |
|  | 10298 | 0 | 0 | 0 | 0 | 0 | 0 |
|  | 10299 | 0 | 0 | 0 | 0 | 0 | 0 |
|  | 10300 | 0 | 0 | 0 | 0 | 0 | 0 |
|  | **Ave:** | **0** | **0** | **0** | **0** | **0** | **0** |

dpc- days post challenge; ave- average; EID_50_- egg infectious dose 50

**Table S3. Ciliary motility scores of SPF chickens 7 days post challenge with live IBV**

|  | **Bird no.** | **Upper** | | | | | **Middle** | | | | | | | **Lower** | | | | | | **Ciliostasis Score^1^** | **APS** | **Total +ve birds** |
| --- | --- | --- | --- | --- | --- | --- | --- | --- | --- | --- | --- | --- | --- | --- | --- | --- | --- | --- | --- | --- | --- | --- |
| **Group A:**  **Live vaccine + VLP vaccine** | 241 | 0 | 0 | | 2 | | | 0 | | 0 | 0 | | 0 | | 1 | | 1 | | 1 | 5 |  |  |
|  | 244 | 0 | 1 | | 0 | | | 0 | | 0 | 0 | | 0 | | 0 | | 1 | | 0 | 2 |  |  |
|  | 245 | 2 | 0 | | 2 | | | 0 | | 0 | 2 | | 2 | | 0 | | 2 | | 2 | 12 |  |  |
|  | 246 | 0 | 0 | | 0 | | | 0 | | 0 | 1 | | 0 | | 1 | | 1 | | 1 | 4 |  |  |
|  | 247 | 0 | 0 | | 0 | | | 0 | | 0 | 0 | | 0 | | 2 | | 2 | | 0 | 2 |  |  |
|  | 248 | 0 | 0 | | 0 | | | 0 | | 0 | 0 | | 0 | | 0 | | 0 | | 0 | 0 |  |  |
|  | 249 | 0 | 0 | | 0 | | | 0 | | 0 | 0 | | 0 | | 2 | | 0 | | 0 | 2 |  |  |
|  | 250 | 0 | 2 | | 0 | | | 0 | | 0 | 0 | | 0 | | 0 | | 0 | | 0 | 2 |  |  |
|  | 252 | 0 | 1 | | 1 | | | 0 | | 0 | 0 | | 0 | | 0 | | 0 | | 0 | 2 |  |  |
|  | 254 | 1 | 1 | | 0 | | | 0 | | 0 | 2 | | 2 | | 2 | | 1 | | 2 | 11 |  |  |
|  |  |  | |  | |  |  | |  | |  |  | |  | |  | |  | |  | **79** | **10/10 (100 %)** |
| **Group B:**  **Live vaccine + Live vaccine** | 264 | 3 | 3 | | 2 | | | 0 | | 0 | 1 | | 0 | | 1 | | 1 | | 1 | 12 |  |  |
|  | 265 | 0 | 0 | | 1 | | | 2 | | 0 | 0 | | 1 | | 1 | | 0 | | 2 | 7 |  |  |
|  | 267 | 1 | 0 | | 1 | | | 1 | | 0 | 0 | | 0 | | 0 | | 1 | | 0 | 4 |  |  |
|  | 270 | 1 | 1 | | 1 | | | 2 | | 2 | 2 | | 1 | | 1 | | 2 | | 2 | 15 |  |  |
|  | 272 | 0 | 0 | | 0 | | | 0 | | 0 | 0 | | 0 | | 0 | | 0 | | 1 | 1 |  |  |
|  | 274 | 1 | 0 | | 1 | | | 0 | | 0 | 0 | | 1 | | 0 | | 0 | | 0 | 3 |  |  |
|  | 276 | 2 | 2 | | 2 | | | 0 | | 0 | 0 | | 1 | | 1 | | 1 | | 1 | 10 |  |  |
|  | 277 | 0 | 1 | | 0 | | | 0 | | 1 | 0 | | 0 | | 0 | | 0 | | 0 | 2 |  |  |
|  | 279 | 0 | 0 | | 0 | | | 1 | | 1 | 0 | | 0 | | 0 | | 0 | | 1 | 3 |  |  |
|  | 280 | 0 | 1 | | 2 | | | 2 | | 1 | 2 | | 1 | | 0 | | 0 | | 0 | 9 |  |  |
|  |  |  | |  | |  |  | |  | |  |  | |  | |  | |  | | **66** | **67** | **10/10 (100 %)** |
| **Group C: Unvaccinated Challenged Control** | 281 | 2 | 3 | | 3 | | | 3 | | 3 | 3 | | 3 | | 2 | | 3 | | 3 | 28 |  |  |
|  | 282 | 2 | 3 | | 3 | | | 3 | | 3 | 3 | | 3 | | 3 | | 3 | | 3 | 29 |  |  |
|  | 283 | 4 | 4 | | 4 | | | 4 | | 3 | 4 | | 4 | | 4 | | 4 | | 4 | 39 |  |  |
|  | 284 | 4 | 3 | | 3 | | | 3 | | 3 | 4 | | 4 | | 3 | | 2 | | 3 | 32 |  |  |
|  | 285 | 3 | 3 | | 3 | | | 3 | | 3 | 3 | | 3 | | 4 | | 4 | | 3 | 32 |  |  |
|  | 286 | 4 | 4 | | 4 | | | 4 | | 4 | 3 | | 3 | | 4 | | 4 | | 4 | 38 |  |  |
|  | 287 | 4 | 4 | | 4 | | | 3 | | 3 | 3 | | 4 | | 4 | | 4 | | 4 | 37 |  |  |
|  | 288 | 3 | 3 | | 3 | | | 3 | | 3 | 3 | | 3 | | 3 | | 3 | | 4 | 31 |  |  |
|  | 289 | 3 | 3 | | 3 | | | 3 | | 4 | 3 | | 3 | | 4 | | 4 | | 3 | 33 |  |  |
|  | 290 | 2 | 2 | | 3 | | | 4 | | 3 | 4 | | 4 | | 4 | | 4 | | 3 | 33 |  |  |
|  |  |  | |  | |  |  | |  | |  |  | |  | |  | |  | | **332** | **-66** | **0/10 (0 %)** |
| **Group D: Unvaccinated Unchallenged Control** | 291 | 0 | 0 | | 0 | | | 0 | | 0 | 0 | | 0 | | 0 | | 0 | | 0 | 0 |  |  |
|  | 292 | 0 | 0 | | 0 | | | 0 | | 0 | 0 | | 0 | | 0 | | 0 | | 0 | 0 |  |  |
|  | 293 | 0 | 0 | | 0 | | | 0 | | 0 | 2 | | 2 | | 2 | | 0 | | 0 | 6 |  |  |
|  | 294 | 1 | 0 | | 0 | | | 0 | | 0 | 0 | | 1 | | 0 | | 0 | | 0 | 2 |  |  |
|  | 295 | 0 | 0 | | 0 | | | 0 | | 0 | 0 | | 0 | | 0 | | 0 | | 0 | 0 |  |  |
|  | 296 | 1 | 3 | | 1 | | | 0 | | 0 | 0 | | 0 | | 0 | | 0 | | 0 | 5 |  |  |
|  | 297 | 2 | 0 | | 4 | | | 0 | | 3 | 0 | | 0 | | 3 | | 4 | | 0 | 16 |  |  |
|  | 298 | 0 | 4 | | 0 | | | 2 | | 0 | 0 | | 0 | | 0 | | 0 | | 0 | 6 |  |  |
|  | 299 | 0 | 0 | | 0 | | | 0 | | 0 | 0 | | 0 | | 0 | | 0 | | 0 | 0 |  |  |
|  | 300 | 0 | 0 | | 0 | | | 0 | | 0 | 0 | | 0 | | 0 | | 0 | | 0 | 0 |  |  |
|  |  |  | |  | |  |  | |  | |  |  | |  | |  | |  | | **29** | **85,5** | **10/10 (100 %)** |

^1^Individual score out of 40; APS- average protection score
